# Draft Genome Assembly of *Neisseria lactamica* Type Strain A7515

**DOI:** 10.1128/genomeA.00951-14

**Published:** 2014-09-25

**Authors:** T. D. Minogue, H. A. Daligault, K. W. Davenport, K. A. Bishop-Lilly, D. C. Bruce, P. S. Chain, O. Chertkov, S. R. Coyne, T. Freitas, K. G. Frey, J. Jaissle, G. I. Koroleva, J. T. Ladner, G. F. Palacios, C. L. Redden, Y. Xu, S. L. Johnson

**Affiliations:** a Diagnostic Systems Division (DSD), United States Army Medical Research Institute of Infectious Diseases (USAMRIID), Fort Detrick, Maryland, USA; b Los Alamos National Laboratory, Los Alamos, New Mexico, USA; c Naval Medical Research Center (NMRC)-Frederick, Fort Detrick, Maryland, USA; d Henry M. Jackson Foundation, Bethesda, Maryland, USA; e Center for Genome Sciences (CGS), USAMRIID, Fort Detrick, Maryland, USA

## Abstract

We present the scaffolded genome assembly of *Neisseria lactamica* type strain A7515 (ATCC 23970) as submitted to NCBI under accession no. JOVI00000000. This type strain of the lactose-fermenting *Neisseria* species is often used in quality control testing and intra-genus phylogenetic analyses. The assembly includes four contigs placed into a single scaffold.

## GENOME ANNOUNCEMENT

*Neisseria lactamica* is a strictly commensal bacterial species originally isolated from a human nasopharynyx. It is unique as compared to other members of the genus *Neisseria* in that it ferments lactose and produces β-galactosidase. *N. lactamica* A7515 (ATCC 23970, NCTC 10617) is the type strain originally described in 1969 (1). This isolate is often used in quality control testing and as outgroup to both *N. gonorrhoeae* and *N. meningitis* in phylogenetic studies (2, 3).

High-quality genomic DNA was extracted from the purified isolate using QIAgen Genome Tip-500 at USAMRIID-Diagnostic Systems Divisions (DSD). Specifically, a 100-mL bacterial culture was grown to stationary phase and nucleic acid extracted as per manufacturer’s recommendations. Sequence data were generated using a combination of Illumina and 454 technologies (4, 5). We constructed and sequenced a 100-bp Illumina library to 312-fold genome coverage and a separate long insert paired-end library (25-fold genome coverage, 7,415±1,854-bp insert, Roche 454 Titanium platform). The two libraries were assembled together in Newbler (Roche) and the consensus sequences computationally shredded into 2-Kbp overlapping fake reads (shreds). The raw reads were also assembled in Velvet and those consensus sequences computationally shredded into 1.5-Kbp overlapping shreds (6). Draft data from all platforms were then assembled together with Allpaths and the consensus sequences computationally shredded into 10-Kbp overlapping shreds (7). We then integrated the Newbler consensus shreds, Velvet consensus shreds, Allpaths consensus shreds, and a subset of the long-insert read-pairs using parallel Phrap (High Performance Software, LLC). Possible mis-assemblies were corrected and some gap closure accomplished with manual editing in Consed (8–10).

Automatic annotation for the *N. lactamica* A7515 genome utilized an Ergatis based workflow at Los Alamos National Laboratory (LANL) with minor manual curation. The annotated genome assembly is available in NCBI, and raw data can be provided upon request. Preliminary review of the 2,181,733-bp (52.2% G+C content) genome found 2,015 coding sequences (CDSs), 12 rRNAs, and 57 tRNAs. One prior assembly of *N. lactamica* ATCC 23970 in 2009 consisted of 101 contigs, but no annotation is provided in NCBI (2). There is one complete genome for the species, *N. lactamica* 020-06, and the annotation statistics are very similar to those seen here (11).

### Nucleotide sequence accession number.

The annotated genome assembly of *Neisseria lactamica* ATCC 23970 is available in GenBank under accession no. JOVI00000000.
